# All that glitters is not gold - founder effects complicate associations of flu mutations to disease severity

**DOI:** 10.1186/1743-422X-7-297

**Published:** 2010-11-01

**Authors:** Raphael TC Lee, Cecília LS Santos, Terezinha Maria de Paiva, Lin Cui, Fernanda L Sirota, Frank Eisenhaber, Sebastian Maurer-Stroh

**Affiliations:** 1 Bioinformatics Institute (BII), Agency for Science Technology and Research (A*STAR), 30 Biopolis Street, #07-01, Matrix, 138671, Singapore; 2 Centro de Virologia, Instituto Adolfo Lutz (IAL). Av. Dr Arnaldo, 355, 01246/902, São Paulo, SP, Brasil; 3 National Public Health Laboratory (NPHL), Ministry of Health (MOH), Singapore; 4 Department of Biological Sciences (DBS), National University of Singapore (NUS), 8 Medical Drive, 117597, Singapore; 5 School of Computer Engineering (SCE), Nanyang Technological University (NTU), 50 Nanyang Drive, 637553, Singapore; 6 School of Biological Sciences (SBS), Nanyang Technological University (NTU), 60 Nanyang Drive, 637551, Singapore

## Abstract

**Background:**

The recent 2009 (H1N1) influenza A pandemic saw a rapid spread of the virus to essentially all parts of the world. In the course of its evolution, the virus acquired many mutations, some of which have been investigated in the context of increased severity due to high occurrences in fatal cases. For example, statements such as: "42.9% of individuals who died from laboratory-confirmed cases of the pandemic (H1N1) were infected with the hemagglutinin (HA) Q310 H mutant virus." give the impression that HA-Q310 H would be highly dangerous or important, while careful consideration of all available data suggests that this is unlikely to be the case.

**Results:**

We compare the mutations HA-Q310 H, PB2-K340N, HA-D239N and HA-D239G using whole genome phylogenetic trees, structural modeling in their 3 D context and complete epidemiological data from sequences to clinical outcomes. HA-Q310 H and PB2-K340N appear as isolated subtrees in the phylogenetic analysis pointing to founder effects which is consistent with their clustered temporal appearance as well as the lack of an immediate structural basis that could explain a change of phenotypes. Considering the prevailing viral genomic background, shared origin of samples (all from the city of Sao Paulo) and narrow temporal window (all death case samples within 1 month), it becomes clear that HA-Q310 H was actually a generally common mutation in the region at that time which could readily explain its increased occurrence among the few analyzed fatal cases without requiring necessarily an association with severity. In further support of this, we highlight 3 mild cases with the HA-Q310 H mutation.

**Conclusions:**

We argue that claims of severity of any current and future flu mutation need to be critically considered in the light of phylogenetic, structural and detailed epidemiological data to distinguish increased occurrence due to possible founder effects rather than real phenotypic changes.

## Background

The problem of founder effects in the analysis of association of viral mutations with clinical phenotypes or fitness of a virus originates from scenarios where initial random mutations are rapidly proliferated in highly connected transmission chains which result in a high occurrence of these founder mutations without the necessity of a selection advantage. In other words, a genetic change common to a small founder population will also be found in most descendants. In viral outbreaks, founder effects can be at play when specific mutations are enriched in samples coming from the same region and same time. Considering phylogenetic relations is useful to identify such viral lineage founder events [1-3] and the perspective of the mutations in protein structures relative to known functional sites is of further help to discuss the possibility of altered phenotypes [3,4]. In the case of the 2009 (H1N1) influenza A pandemic, some mutations have received particular attention due to their apparent increased occurrence in severe cases. The best studied, HA-D239G, is also referred to in the literature as D222G or D225G using alternative (e.g. seasonal H1/H3) numberings. Although generally rare, HA-D239G was found by several groups to appear enriched in severe cases [5-9]. The conservative WHO estimate suggested that this mutation was found in 7% of all global death cases [9]. The same WHO report also mentions HA-D239N and PB2-K340N as being under investigation but with unknown clinical significance [9]. A separate study suggested HA-D239G and HA-Q310 H to be associated with disease severity noting that "42.9% of individuals who died from laboratory-confirmed cases of the pandemic (H1N1) were infected with the hemagglutinin (HA) Q310 H mutant virus" [10].

Here, we analyze the phylogenetic distribution and structural positioning of HA-Q310 H, PB2-K340N, HA-D239N and HA-D239G to identify possible biases through founder effects and further discuss the mutations in their structural context and temporal appearance.

## Methods

Naturally occurring pandemic 2009 (H1N1) influenza A viral sequences that were submitted between 30 March 2009 to 31 May 2010 were downloaded from the NCBI Influenza Virus Resource [11]. A total of 3588 viral strains were analyzed. The sequences for each protein were aligned with MAFFT [12] and substitutions in positions of all 10 proteins for all 3588 strains were identified relative to reference strain A/Texas/04/2009 as it was one of the first submitted strains with sequence information available for all viral genes that most closely resembles the rest of the circulating H1N1 strains during the first week of sequence submission. Phylogenetic analysis was conducted on all strains with full-length nucleotide sequences available for all 8 segments. The protein coding nucleotide sequences for these strains were concatenated such that a single sequence representing a single strain contains nucleotides for all 10 proteins. These sequences were aligned with MAFFT [12] using the FFT-NS-1 option. Cd-hit [13] was used to remove highly similar sequences by allowing a maximal sequence identity of 99.94% to reduce the set to 727 non-redundant strains. Next, we created a maximum likelihood tree using PhyML [14] with the approximate likelihood ratio test, the HKY85 substitution model and other parameters such as for the shape of the gamma distribution (0.353) were estimated by the program. The major strain lineages and substitutions discussed in this analysis were identified and marked in the resulting phylogenetic tree using the MEGA 4 package [15].

To assess the overall extent of clustering for the mutations of interest in the phylogenetic tree, we computed the association index (AI) [16], parsimony score (PS) [17] and the monophyletic clade (MC) size statistics using BaTS (Bayesian tip-association significance testing) [18]. The BaTS program examines a posterior sample of trees generated by a Bayesian Markov Chain Monte Carlo (MCMC) approach implemented in BEAST v1.6.0 (Bayesian Evolutionary Analysis Sampling Trees) [19]. The input alignment was the same as described above. The mean substitution rate was estimated using the HKY substitution model, a strict molecular clock, and a constant population size coalescent prior. A chain length of 10 million generations was performed to ensure that all parameters had achieved stabilization, as assessed by the program TRACER v1.5.0 http://tree.bio.ed.ac.uk/software/tracer/. The posterior trees were sampled every 1,000 generations and the maximum likelihood tree generated from PHYML was used as a starting tree. The BaTS program is then performed with 1,000 replications and with the first 1,000 sampled trees removed as burn-in.

To observe the emerging trends of the substitutions HA-K2E, HA-Q310 H, PB2-K340N, HA-D239N and HA-D239G, the number of strains carrying these 5 substitutions was recorded according to their collection date. A window period of 28 days was used to estimate the average percentage of observing a particular substitution, over the total number of strains with sequence information at the position of the substitution. Since the first sample collected falls on 30 March 2009, the first data point in the percentage-time graph, which represents an average percentage of the substitution over the past 28 days, will be on 26 April 2009. As there are relatively much fewer sequences available from February 2010, inclusion of data from this date forward will result in unreliable fluctuations. Hence, data points from February 2010 onwards were not included in this percentage-time graph analysis.

The structural mapping of the mutations is based on the crystal structure of 2009 H1N1 hemagglutinin (PDB: 3LZG) [20] modeled with a human host cell receptor analogue (LSTC) as well as a homology model of polymerase basic protein 2 using PDB: 2VQZ[21] as template. Modelling and visualization of structures was done with Yasara [22].

Sequencing methodology (by Instituto Adolfo Lutz): Viral RNA was extracted either from clinical samples or supernatant fluid from MDCK infected cells using the QIAmp Viral RNA Extraction Kit (QIAGEN, Valencia, CA, US) according to the manufacturer's instructions. For viral RNA extraction from necropsy tissues the QIAmp Blood Viral RNA Extraction Kit was used instead. Primers designed to amplify the complete HA gene sequence as well as the RT-PCR amplification and sequencing protocols were those provided by WHO http://www.who.int/csr/resources/publications/swineflu/sequencing_primers/en/index.html. RT-PCR products were directly sequenced using the "ABI Prism Big Dye Terminator Cycle Sequencing Ready Reaction kit (PE Applied Biosystems, Foster City, CA, US), Sequences were determined in an Applied Biosystems 3130 ABI Genetic Analyzer. The following sequences were deposited in GenBank under accession numbers: GQ247724; GQ356787; GQ368664-GQ368667; GQ414764-GQ414768; GQ915017-GQ915025. Accessions of sequences discussed in detail are indicated in the main text.

## Results and Discussion

**Figure 1:**
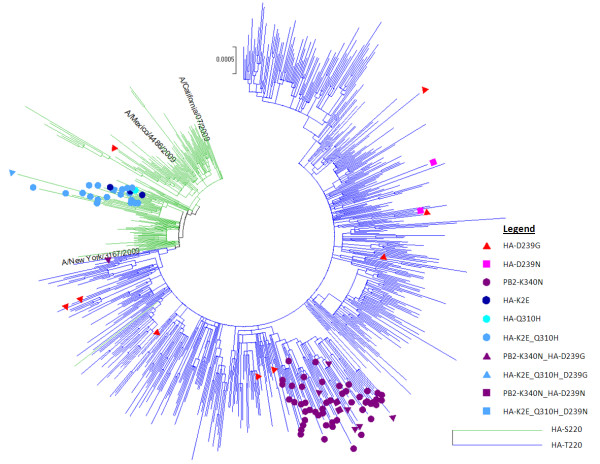
**Whole coding genome maximum likelihood phylogenetic tree with viral strains labeled according to mutations of interest to distinguish independent and cluster occurrences**.

Our whole coding genome maximum likelihood phylogenetic tree of 2009 (H1N1) influenza A strains (Figure 1, see also Materials and Methods) is in good agreement with previous studies [2,23,24]. We see early diversification into clades from Mexico and California which are superseded by a dominant clade (corresponding to clade number 7 in Nelson *et al*. and Valli *et al*.) that further evolves with characteristic marker mutations (e.g. HA S220T). Also, the known time stamps of samples follow the phylogenetic groupings and approximate order in the tree. When comparing the distribution of the mutations of interest, we see clearly distinct patterns where HA-Q310 H and PB2-K340N are each confined to monophyletic clusters suggestive of founder effects while HA-D239G is not restricted to a monophyletic group but rather occurred several times independently in strains that are not closely related and is, hence, not likely to associated with founder effects. Only few strains are available with HA-D239N, however, they seem to resemble more closely the scattered distribution of HA-D239G.

**Figure 2:**
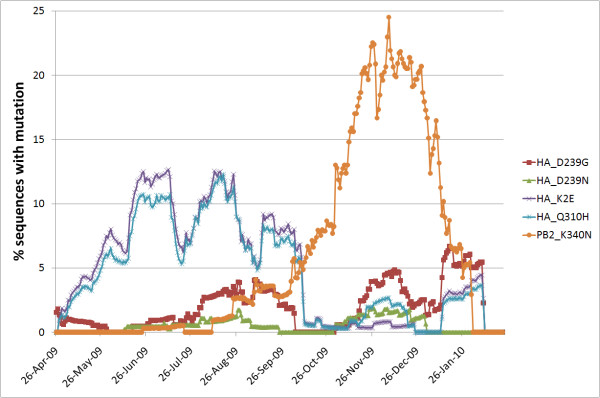
**Temporal global appearance of mutations of interest shown as 28 days sliding window average of % sequences with the respective mutation**.

This phylogenetic clustering is further supported by the temporal global appearance of the mutations, with HA-Q310 H and PB2-K340N being predominantly restricted to continuous time periods whereas HA-D239G independently re-occurred several times during the pandemic (Figure 2).

**Figure 3:**
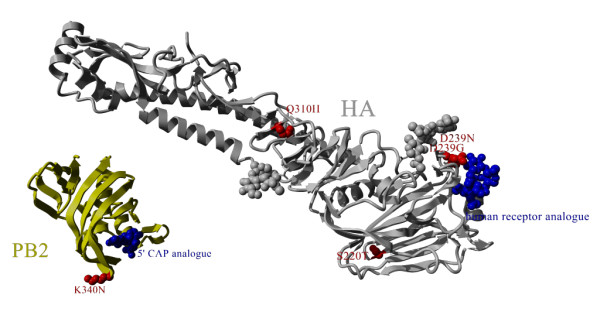
**Positions of discussed mutations in viral protein structures of hemagglutinin (HA) and polymerase basic protein 2 (PB2)**. Only HA-D239G and HA-D239N appear in a position that can be rationalized to directly alter the phenotypic properties of the virus.

Indeed, although HA-D239G is generally rare, it was found in 7% of all global death cases [9]. It has also been shown to alter the biology of the virus [25] and may be rationally associated with severity by switching host cell receptor specificity (Figure 3). HA-D239N has also been found with increased incidence in severe cases [7] and could similarly affect the host cell recognition properties. HA-Q310 H, on the other hand, is located far from the receptor binding pocket and no direct biomolecular mechanism is known yet for this position that could support a change in severity (Figure 3).

The same applies to PB2-K340N which is at a structural position where the mutated sidechain is pointing away from the functionally important cap binding site of the PB2 5' cap snatching domain (Figure 3). The mutation is not in close vicinity (within 5 Angstroem) of PB1 or PA in any currently known structure. 8 of 123 strains with PB2-K340N and HA sequence information in Genbank also have the HA-D239G mutation. These 8 cases are strongly biased in their geographic occurrence with 7 coming from Russia. PB2-K340N is being investigated for its occurrence in severe cases [9] but given its temporal (Figure 2), geographical and phylogenetic (Figure 1) clustering, it could be that this higher incidence may be associated to founder effects rather than direct phenotypic changes.

The particular case of disease severity association of HA-Q310 H was proposed by Glinsky [10] based in part on the analysis of 7 Brazilian fatal cases where 3 of them had the HA-Q310 H mutation (GenBank:GQ414768, GenBank:GQ915019, GenBank:GQ915020), 2 had the HA-D239N mutation (GenBank:GQ915021, GenBank:GQ915020 [also has HA-Q310H]) and 2 had the HA-D239G mutation (GenBank:GQ915017, GenBank:GQ915018). Here, we add the clinical information for two more Brazilian cases with HA-Q310H: one more fatal case (GenBank:GQ915025) as well as a mild case (GenBank:GQ368664). The existence of mild cases with this mutation is important as it shows that the individual outcome depends on additional patient-specific factors. HA-Q310 H also occurred in two Singaporean samples (GenBank:CY049659, GenBank:CY049284)[26] for which clinical information is available and both cases were mild.

**Figure 4:**
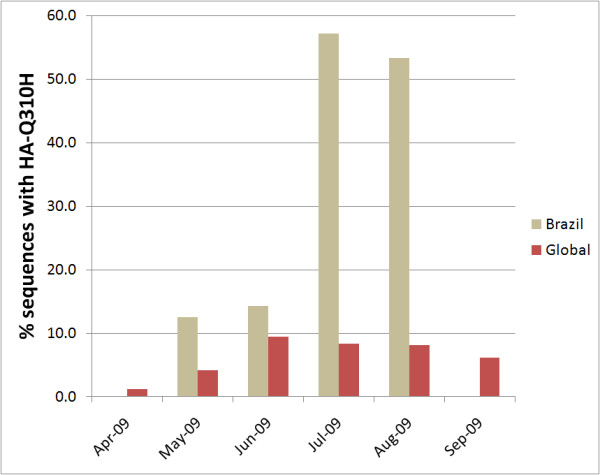
**Average monthly percentage of strains with the HA-Q310 H mutation compared between Brazil and the whole world**. Clearly, HA-Q310 H appeared more frequently in Brazil in the months July and August which was the exact time frame of the analyzed death cases.

In total, 18 out of 47 Brazilian HA sequences (38%) were found to have the Q310 H mutation, while globally this applied to only 160 out of 3240 HA sequences (5%) over the timeframe analyzed (Apr 2009 - Jan 2010). Although HA-Q310 H occurred in 4 of the 8 (50%) Brazilian death cases from July and early August, this is similar to its overall occurrence in Brazil at that time (53-57%, Figure 4). Considering the prevailing viral genomic background, shared origin of samples (all 8 from the city of Sao Paulo) and narrow temporal window (all death case samples within 1 month), it becomes clear that HA-Q310 H was actually a generally common mutation in the region at that time which could readily explain its increased occurrence among these analyzed cases due to founder effects without requiring necessarily an association with severity.

Additionally, it was noted [10] that HA-Q310 H co-occurred with a specific genotype on position HA 220. Indeed, HA-S220T has already been recognized as typical marker mutation of different phases of the outbreak likely due to founder effects (Figure 1)[2,27,28]. Further analysis of the strains with HA-Q310 H reveals that most of them also contain another mutation, HA-K2E, which was therefore also included in the analysis. Indeed, 3 of the 4 fatal Brazilian cases with HA-Q310 H also had HA-K2E. Again, due to its close phylogenetic clustering, almost exclusive co-occurrence with HA-Q310 H (Figure 1) as well as strong temporal correlation with HA-Q310 H (Figure 2), HA-K2E likely appeared more frequently during that period due to founder effects rather than being phenotypically important.

Finally, in order to estimate the statistical significance and quantify the extent of phylogenetic clustering of the discussed mutations, we computed the association index (AI) [16], parsimony score (PS) [17] and the monophyletic clade (MC) size statistics using BaTS (Bayesian tip-association significance testing) [18] over a posterior sample of trees generated by BEAST [19]. As shown in Table 1, the AI and PS index ratios of the mutations HA-D239G and HA-D239N approach 1. This confirms their sporadic and clade-independent nature in the phylogeny. On the other hand, the index ratios and monophyletic clade size statistics indicated that the 3 mutations HA-K2E, PB2-K340N and HA-Q310 H were more likely to be associated with founder effects as compared to the other 2 mutations.

**Table 1:** BaTS results for the extent of clustering of selected mutations in the phylogeny.

Statistic	Mutation	Index Ratio, observed to expected (95% CI)	Observed Value (95% CI)	Expected Value (95% CI)	P-value
AI	HA-D239G	0.74 (0.50-1.11)	2.75 (2.22-3.26)	3.70 (2.95-4.46)	0.03
AI	HA-D239N	0.63 (0.29-1.37)	0.79 (0.49-1.07)	1.25 (0.78-1.67)	0.06
AI	HA-K2E	0.07 (0.05-0.10)	0.39 (0.34-0.46)	5.30 (4.40-6.21)	0
AI	PB2-K340N	0.03 (0-0.04)	0.31 (0.05-0.41)	11.28 (9.87-12.67)	0
AI	HA-Q310H	0.07 (0.01-0.17)	0.31 (0.06-0.64)	4.71 (3.81-5.60)	0
PS	HA-D239G	0.95 (0.94-1)	17 (17-17)	17.87 (17-18)	0.07
PS	HA-D239N	0.84 (0.83-0.83)	5 (5-5)	5.99 (5.99-6)	0.01
PS	HA-K2E	0.12 (0.12-0.12)	3 (3-3)	25.72 (24.87-26)	0
PS	PB2-K340N	0.07 (0.07-0.07)	4 (4-4)	56.51 (54.60-57.87)	0
PS	HA-Q310H	0.18 (0.17-0.18)	4 (4-4)	22.79 (22-23)	0
MC	HA-D239G	NA	2 (2-2)	1.12 (1-2)	0.06
MC	HA-D239N	NA	2 (2-2)	1.01 (1-1.01)	0.01
MC	HA-K2E	NA	24 (24-24)	1.25 (1-2)	0
MC	PB2-K340N	NA	17.32 (17-18)	1.82 (1.12-2.23)	0
MC	HA-Q310H	NA	6.54 (4-11)	1.20 (1-2)	0

## Conclusions

The interpretation of effects of mutations on observed patient phenotypes is notoriously difficult. Besides underlying conditions of the patient, factors as simple as delayed admission to hospital may have a strong influence on disease outcome. Often, only one or two genes of each patient's viral strain but not the complete genome sequence are available, and hence the full spectrum of mutations for each case is rarely known, though this is changing now with the advent of new sequencing technologies. Overrepresentation of certain mutations among geographically and temporally related samples needs to be carefully controlled for possible founder effects which could be identified as homogenous clusters in phylogenetic analyses, as was observed to be the case for HA-Q310 H and PB2-K340N but not HA-D239N or HA-D239G. While founder effect mutations cannot automatically be linked to phenotypes simply by increased occurrence, they may nevertheless alter the virus fitness for which even tiny changes could result in advantages shifting selection to their favor. This, however, requires thorough experimental testing and careful consideration of their structural roles and phylogenetic relationships. Another problem is the natural bias of sequencing severe cases disproportionally more than mild cases. Therefore, a seemingly high percentage of a mutation among severe cases needs to be viewed in the context of the general viral genomic background in the same region and time frame as the samples.

## Abbreviations

HA: hemagglutinin; PB2: Polymerase basic protein 2.

## Competing interests

The authors declare that they have no competing interests.

## Authors' contributions

CLSS, TMP and LC coordinated and executed sample collection, molecular sequencing and the epidemiological and clinical analysis. RTCL and SMS carried out the phylogenetic analysis. FLS and SMS contributed the structural analysis. RTCL, FLS, FE and SMS conceived and designed the study and drafted the manuscript. All authors read and approved the final manuscript.
